# TFE3 is a Novel Biomarker of Ovarian Sclerosing Stromal Tumours

**DOI:** 10.21203/rs.3.rs-2563971/v1

**Published:** 2023-02-13

**Authors:** Li Zhao, Yan Zhou, Yuping Liu, Qiuping Luo, Qingping Jiang, Hui Wang, Na Wang

**Affiliations:** The Third Affiliated Hospital of Guangzhou Medical University; Affiliated Cancer Hospital & Institute of Guangzhou Medical University; The Fourth Affiliated Hospital of Guangzhou Medical University; The Third Affiliated Hospital of Guangzhou Medical University; The Third Affiliated Hospital of Guangzhou Medical University; Affiliated Cancer Hospital & Institute of Guangzhou Medical University; The Third Affiliated Hospital of Guangzhou Medical University

**Keywords:** Ovarian Sclerosing Stromal Tumours, TFE3, Novel Biomarker

## Abstract

Sclerosing stromal tumours of the ovary are benign and tend to occur in young women with lobular structures at low frequencies. Three types of cells, luteinized cells, short spindle myoid cells, and intermediate cells, are found in lobules, which are rich in blood vessels. Currently, immunohistochemistry and fluorescence in situ hybridization are used to detect normal follicles, sclerosing stromal tumours, granulosa cell tumours, and theca fibromas. Our research found the expression of transcription factor enhancer 3 (TFE3) was moderately and strongly positive in the inner thecal cell layer of normal follicles. It was expressed in seven out of eight sclerosing stromal tumours, mainly in luteinized cells, but not in 20 granulosa cell tumours and 1 microcystic stromal tumour. In nine cases of theca cell tumours and theca fibromas, TFE3 was not expressed, except in two cases of weak TFE3 expression. Eight cases of sclerosing stromal tumours were analysed by FISH using a TFE3 separation probe, and the results were negative. In a word, as a nuclear transcription protein, TFE3 was specifically expressed in sclerosing stromal tumours and could serve as a new marker for the diagnosis and differential diagnosis of sclerosing stromal tumours.

## Introduction

Sclerosing stromal tumour (SST) is a rare sex cord-stromal ovarian tumour that was first reported by Chalvaridjian and Scully in 1973 ^[1]^. SST occurs predominantly in young women of 20–30 years of age ^[2, 3]^, and its clinical symptoms include pelvic pain, menstrual irregularity ^[4]^, and nonspecific symptoms associated with ovarian cysts. In a few cases, patients had elevated serum levels of CA125 ^[5]^. However, the levels of hormones in these patients were not affected. Focal adenoid hyperplasia was diagnosed in only 1 of the 10 cases, but endometrial biopsy could not be performed in this case ^[5]^. In addition, a case of ovarian SST complicated by endometrial adenocarcinoma has been reported ^[6]^.

Characteristic feature of SST is the abundance of blood vessels in the nodules. Computed tomography (CT) and magnetic resonance imaging (MRI) have been employed to diagnose SSTs, particularly to assess their vascularization ^[7–9]^. New ultrasound technologies have been developed to facilitate the exploration of adnexal masses, such as the diagnosis of tissue vascularization via colour Doppler ^[10]^. This blood flow feature of SST is relatively unique, which is helpful to differentiate from other sex cord stromal tumors and ovarian malignant tumors. This method not only obtains examination results quickly but also reduces the economic burden on patients.

In general, SST is unilateral, mostly 5–10 cm, with well-defined boundaries and sometimes a thin fibrous envelope. It is a grey–white to grey–yellow nodular oedema, and unilocular cystic cases are rare ^[11]^. According to previous studies, SST consists of three types of cells, lipid-rich cells, fibroblast-like cells, and undifferentiated stromal mesenchymal cells, with intermediate morphology showing different degrees of differentiation ^[12]^. In addition, some studies indicate that tumour cells in SST have the characteristics of muscle-like cells and express SMA or desmin ^[13, 14]^. Growing evidence shows that cytoplasm-rich cells express calretinin and inhibin ^[15]^ but do not express SMA, desmin, CK, and CK7 ^[16 18]^. In some cases, lipid-rich cells express CD10 ^[19]^ and Melan A ^[20, 21]^. These cells are similar to normal cells in the ovary but have not been clearly defined.

A study found that TFE3 was highly expressed in sclerosing stromal tumors and pointed out TFE3 is highly expressed in the nucleus of lutein cells and polygonal-to-round tumour cells in 7 out of 9 patients with SST ^[22]^. However, neither luteinized fibromas nor thecomas express appreciable levels of TFE3 ^[22]^. Fluorescence in situ hybridization (FISH) analysis revealed the presence of trisomy 12 in >20% of SST cells ^[12]^. In 2020, using whole-exome, targeted capture, and RNA sequencing, Sarah et al. reported that 65% (17/26) of SST patients had recurrent FHL2-GLI2 fusion genes and 15% (4/26) had other GLI2 rearrangements ^[23]^. Specifically, these genetic abnormalities were not detected in other types of sex cord-stromal tumours (n = 48) and common cancers (n = 9,950).

In this study, the origin of SSTs was discussed. TFE3 immunohistochemical and molecular analyses were performed on SSTs and other types of ovarian sex cord-stromal tumours. TFE3 staining was used to distinguish SST from other sex cord-stromal tumours.

## Materials And Methods

### Patients

The records of 38 patients who had undergone surgical resection of sex cord-stromal ovarian tumours at The Third Affiliated Hospital of Guangzhou Medical University (Guangzhou, China) between December 2013 and December 2021. The focus of this study is sclerosing stromal tumor (8 cases), and its main differential diagnosis is also involved, which are theca cell tumor and theca fibroma tumour (9 cases), granulosa cell tumour (20 cases), and microcystic stromal tumour (1 case)in turn. The age of patients ranged from 17 to 70 years and they had not received any pre-operative therapy.

Ethical approval was obtained from the institutional review board of the ethics committee.

### Lipid stains (Oil Red O)

The biopsy samples were placed in a tissue tek container (Sakura Finetek, CA, USA) and then filled with tissue tek OCT compound gel. After being cut into 7-μm slices, the samples were snap-frozen in liquid nitrogen and stained with Oil Red O according to standard procedures.

The oil red O ^24^ fat staining method is usually used to detect the fat in tissues or cells. Oil red O is a fat-soluble dye, which is a strong fat solvent and fat dye, and can be highly dissolved in fat. Its dyeing principle is that oil red O can specifically adsorb with the neutral triglycerides, lipids and lipoproteins in tissues and cells to make fat dye. The solubility of dye in intracellular lipids is greater than that of solution.

### Immunohistochemistry (IHC)

Collected tumours or normal tissue after removal of paraffin as the experimental subject. Consecutive 4-μm thick unstained sections for immunohistochemical staining, which was performed using the Leica automatic immunostaining device (Leica Microsystems, Inc.). Primary antibodies against CD10 (1:100; no. 563871; DAKO; DK), α-inhibin (1:100; no. GT230202; CHN), SMA (1:50; no. MAB-0980; MXB; CHN), desmin (1:300; no. GT225202; Gene tech; CHN), TFE3 (1:100; no. ZA-0657; ZSGB-BIO; CHN), calretinin (1:100; no. ZM-0063; ZSGB-BIO; CHN), WT-1 (1:100; no. ZM-0269; ZSGB-BIO; CHN), and EMA (1:300; no. GM061302; Gene tech; CHN). Appropriate positive and negative controls were simultaneously stained to validate the staining method.

### Immunohistochemistry were conducted according to previously described methods.

All slides were reviewed and scored independently by three pathologists. The pathologists were blinded to the experiment. The scoring method based on both the intensity (0, no staining; 1 +, weak staining; 2+, medium staining; 3+, strong staining).

### Fluorescence in Situ Hybridization (FISH) analysis

The TFE3 isolation probe was provided by Guangzhou LBP Medicine Science and Technology Co., Ltd. (China). Specific operations were performed according to the manufacturer’s protocol. The results showed that there was no fracture of TFE3 gene in females (2 yellow) and males (1 yellow). In females, 1 red 1 green 1 yellow, and in males, 1 red 1 green indicated that TFE3 had a balanced translocation and the gene was fused. In females, 1 red and 2 yellow, and in males, 1 red and 1 yellow, showed unbalanced translocation and fracture of TFE3 gene.

## Results

### Clinical findings

As shown in Table 1, the age range of patients with SSTs was 17–39 years old. The levels of hormones were normal in all patients. However, menstruation was irregular in cases 5 and 7. In case 5, an adnexal mass was found during physical examination, and no abnormality was found in the endometrium. Endometrial biopsy showed atypical hyperplasia, and an adnexal mass was found in case 7. In case 4, in which the patient presented with abdominal distension for 1-year, an adnexal tumor was found on B-ultrasound with peritoneal effusion. Adnexal tumours were found in other patients during physical examinations.

### Gross Findings

**Figure 1:**
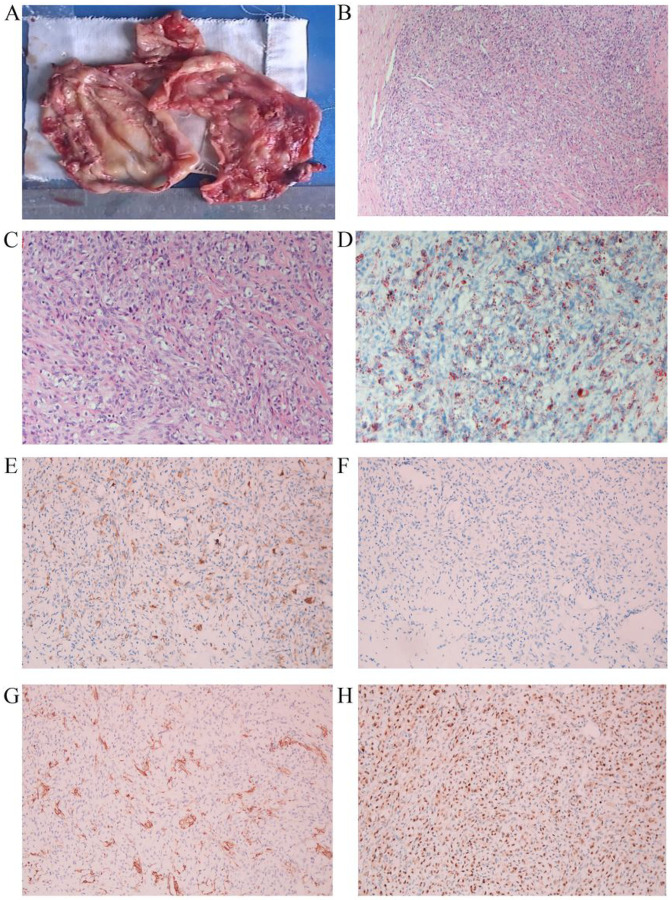
One cystic case of SST. The cut surface of SST was cystic (A). The cellular foci were consistedof roundcells admixed with spindle cells (B and C). Oil Red Ostaining was positive in lipid-rich cells (D). The tumor cells weakly expressed inhibin (E). The round cells and spindle cells did notexpress desmin (F). The short spindle cells in the tumor expressingSMA (G). TFE3 was diffuse positive in the lipid-rich cells (H).

**Figure 2:**
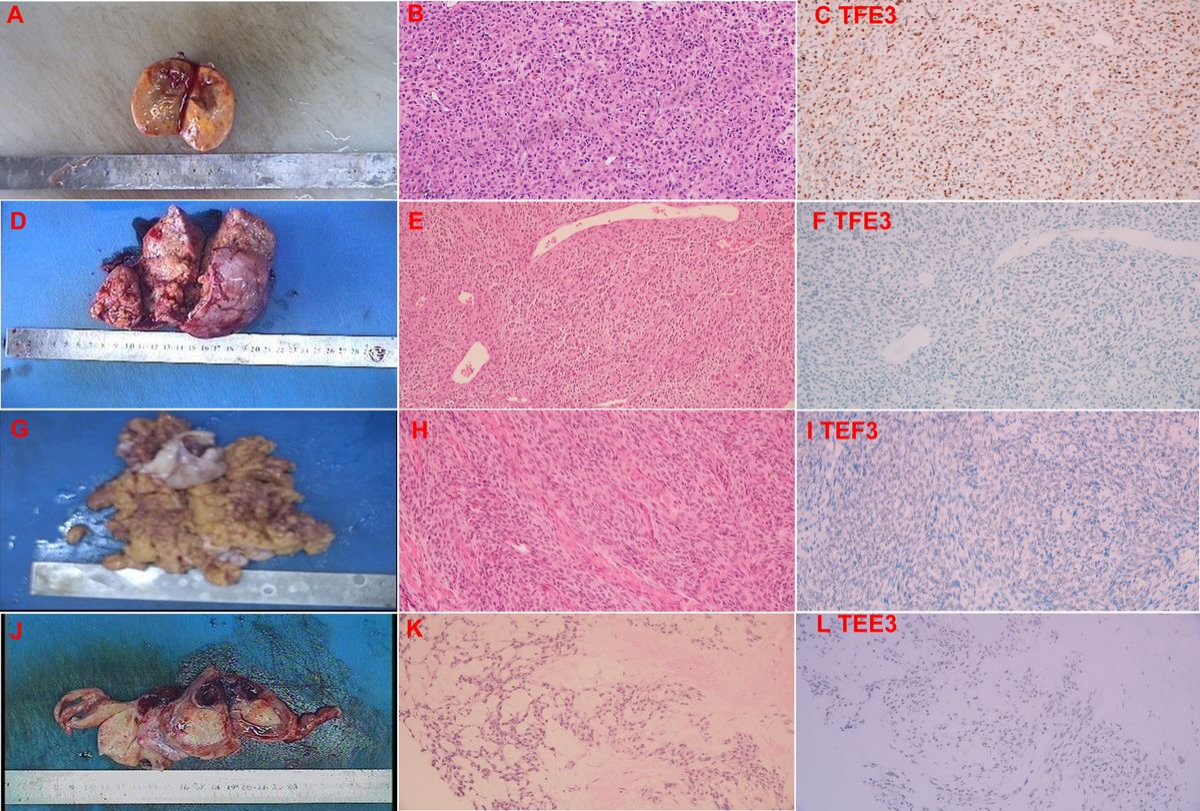
Expression of TFE3 in different types of sex cord stromal tumours. The cut surfaces of SST were solid with yellow (A). The cytoplasm of most cells in the lobules is rich, eosinophilic and granular (×200) (B). Cytoplasm-rich cells express TFE3 (C). The cut surface of the adult granulosa cell tumor is nodular and light yellow (D). Granulosa cells usually have scanty cytoplasm and pale, uniform, angular to oval, often grooved nuclei that are typically arranged haphazardly to each other, and typical Call-Exner bodies can be seen (E). TFE3 is not expressed in AGCT (F). Thecoma. The typical sectioned surface of a thecoma showing a yellow appearance (G). Thecoma. A high-power view shows the characteristic appreciable pale grey cytoplasm, ill-defined cytoplasmic membranes, and scattered collagen bundles (H). TFE3 is not expressed in the tumor cells of thecoma (I). Microcystic stromal tumor. The sectioned surface showing a solid, yellow appearance (J). Microcystic stromal tumor. Characteristic small cysts and hyaline plaques are seen (K). The tumor cells do not express TFE3 (L).

The size of the eight tumours ranged from 14 to 160 mm. All tumours were well-circumscribed nodules, and except for one cystic case (Fig. 1A), all tumours were solid (Fig. 2A), which ranged from soft to tough. The cut surfaces of the solid tumours were typically white, slightly leafy, with scattered yellow nodules and were most abundant at the periphery. Focal haemorrhage was observed in one patient. In case 1, the tumour was cystic, with cysts containing thick gelatinous material (Fig. 1A).

### Histologic Findings And Immunohistochemical Results Of Sclerosing Stromal Tumours

All tumours, including cystic tumours, showed overt, often discrete, cellular and hypocellular regions, resulting in a pseudolobular appearance (Fig. 1B). The cellular areas and lower cellular intervals were always collagenous, loose collagenous, and markedly oedematous. Cellular foci consisted of a mixture of round and spindle cells (Fig. 1C). The cytoplasm of the former was pale, eosinophilic, vacuolated, or foamy to varying degrees (Fig. 1C). Thin-walled, dilated, focally branching (“staghorn”) blood vessels were prominently found in all cases, and markedly conspicuous labyrinthine and haemangiopericytoma-like tumours were evident in some cases (Fig. 2B). Oil Red O staining revealed that the cytoplasm was rich in lipids but not in mucus (Fig. 1D). Spindle cells expressed SMA (Fig. 1E) but not desmin (Fig. 1F). CD34 staining showed abundant blood vessels in the cell lobules (Fig. 1G). Only cells with abundant cytoplasm in the tumours expressed TFE3, and nuclear expression was moderately and strongly positive (Fig. 1H).

### Expression Of Tfe3 In Ovarian Sex Cord Stromal Tumours

As shown in Table 2, TFE3 was highly expressed in sclerosing stromal tumours (7/8), weakly expressed in two cases of thecoma (2/9), and not expressed in other tumours (0/21, including 20 cases of granulosa cell tumours and one case of microcystic stromal tumours). As shown in Fig. 2, TFE3 was mainly expressed in sclerosing stromal tumours (Fig. 2A, B, C) but not in ovarian granulosa cell tumours (Fig. 2D, E, F), thecoma (Fig. 2G, H, I), or microcystic stromal tumours (Fig. 2J, K, L). Moreover, TFE3 mainly expressed in the nucleus of cells with abundant cytoplasm, and the staining intensity was medium to strong (Fig. 2C).

### Immunophenotypes Of Theca Cells In Normal Follicles

**Figure 3:**
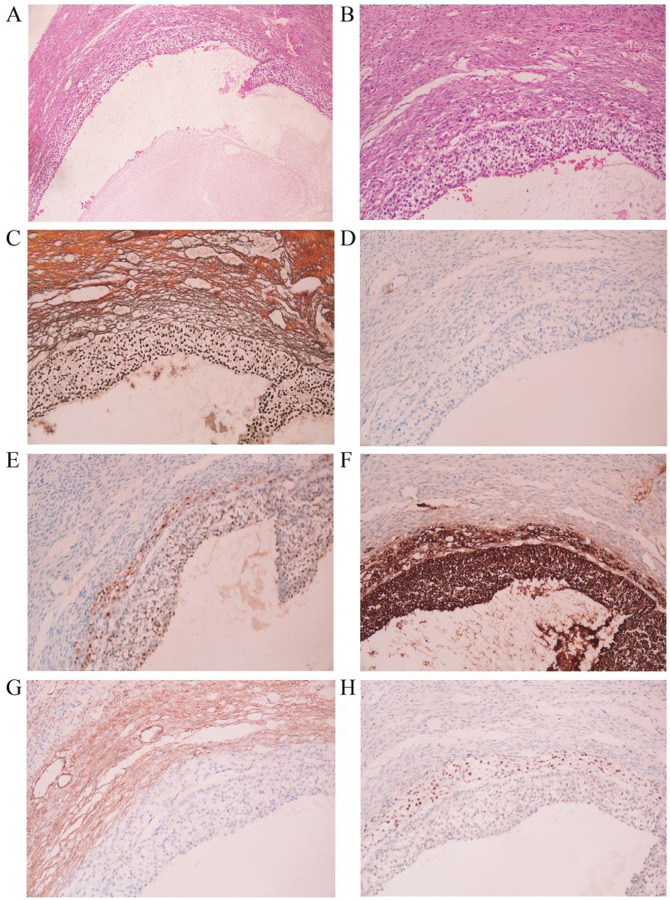
Normal follicular tissue. The follicle was observed at low magnification (×40) (A) and high magnification (×200) (B). Reticular fibers surrounded the inner theca cells and were absent around granulosa cells (C). CD10 was negative (D). The inner theca cells and granulosa cells expressed calretinin and inhibin (E and F). SMA was mainly expressed in the outer theca cell layer (G). TFE3 was expressed in the inner theca cells (H).

No literature has reported the expression mode of TFE3 in normal ovary. In order to explore the source of TFE3 immunohistochemistry positive cells in sclerosing stromal tumors, we collected 10 normal ovarian tissues. Normal ovarian follicles were obtained from specimens removed due to ovarian endometriosis or teratoma. We tested a total of 30 follicles. Normal follicular structure was observed at low and high magnifications. The boundary between the inner and outer theca cell layers was not very clear, but the cytoplasm of inner theca cells is rich and clear (Fig. 3A and B). A mostly consistent immunophenotype was observed in the rich cytoplasm cells: each demonstrated diffuse TFE3, TFE3 expressed in the inner theca cells, but not in the granular layer, outer theca layer, or fibrous tissue in normal follicles (Fig. 3H).

#### FISH detection

**Figure 4:**
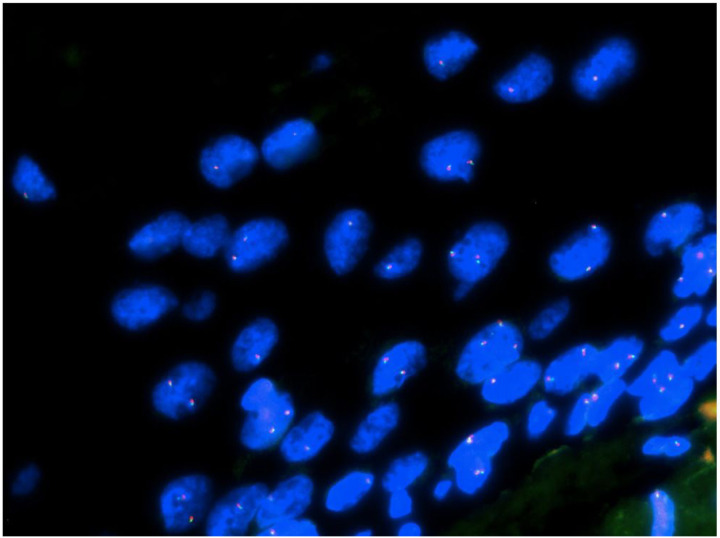
The results of FISH. There was no separation of TFE3 in SST.

Using the separation probe of TFE3, TFE3 was detected by FISH in 7 cases of SSTs expressing TFE3. The results showed no separation of TFE3 in these seven cases (Fig. 4).

## Discussion

TFE3 is located at Xp11.23, and its protein belongs to the microphthalmia-associated transcription factor (MiTF) family, which plays an important role in the regulation of lysosomal biogenesis and autophagy ^[25]^. The TFE/MiTF family consists of four important members: (i) TFEB, (ii) TFEC, (iii) TFE3, and (iv) MITF ^[26]^. By searching the human protein atlas webtool (https://www.proteinatlas.org/), TFE3 was found to be expressed in adipose tissue, urinary bladder, ovary, testis, breast, etc. TFE3 protein can be transported to the nucleus when cells are starved and/or stressed. The expression and activity of TFE3 are upregulated in many types of human cancers, and are associated with enhanced proliferation and motility of cancer cells. The main tumours related to TFE3 gene fusion include epithelioid haemangioendothelioma, alveolar soft-part sarcoma (ASPS), renal cell carcinoma, malignant chondroid syringoma, rare ossifying fibromyxoid tumours, and perivascular epithelioid cell tumor ^[27–34]^.

Park CK and Kim HS had reported that TFE3 was expressed in sclerosing stromal tumors, but there was no abnormality in TFE3 gene ^[22]^. Our research got the same result that TFE3 was specifically expressed in sclerosing stromal tumours, but not in other sex cord stromal tumours. Moreover, TFE3 was specifically expressed in luteinized cells, but not in other two cells.

Sclerosing stromal tumours often occur in young women, and a few cases have the secretion of estrogen and/or androgen. The clinical results of all cases were benign. This study analyzed 8 cases of sclerosing stromal tumours, ranging in age from 17 to 39 years old. No hormone abnormality was found clinically, but 2 patients had irregular menstruation. Follow-up results showed no recurrence.The lobulated structure and three types of cell composition are the unique morphological characteristics of ovarian sclerosing stromal tumours. Due to the young age of SST and good prognosis, accurate pathological diagnosis is very important in order to avoid over-treatment. In most cases, we can obtain a positive pathological diagnosis based on the above findings. However, due to the diversity and variety of ovarian sex cord stromal tumours, we need more specific indicators to assist the pathologist in diagnosis in a few cases.For example, the cystic case in Fig. 1.

Our experimental results show that the luteinized tumor cells in sclerosing stromal tumors have abundant intracellular lipids, and Oil red O staining is obvious. Although microencapsulated stromal tumors and granulosa cell tumors have no intracellular lipids, and Oil red O is negative, there can also be intracellular lipids in thecal cell tumors. Meanwhile, there was no significant difference in immunohistochemical results between SSTs and other ovarian sexual cord stromal tumours, because SF-1, calretinin and inhibin were also expressed in these tumours. Therefore, the positive expression of TFE3 immunohistochemistry is of great significance in the diagnosis and differential diagnosis of sclerosing stromal tumors ^[5,7,11,14–16]^

Because there is no abnormality of TFE3 gene in sclerosing stromal tumors, in order to explored the source of TFE3 positive cells, we analyzed the expression of TFE3 in normal ovaries. To our knowledge, this is the first study to demonstrate that TFE3 nuclei are expressed in the inner membrane cells of follicles, but not in the granulosa cell layer, outer membrane cell layer, or fibroblasts. However, it remains unclear whether the nuclear localization of TFE3 is involved in egg maturation and plays a role in intrathecal cells, which will be the focus of our next study.

In our study, seven out of eight cases of SSTs expressed luteinized cells with moderate-to-severe staining of TFE3, but it was not expressed in the ovarian granulosa, theca, or microcystic stromal tumor. Moreover, the FISH analysis revealed that the TFE3 gene was not broken, indicating that there was no possibility of TFE3 fusion with other genes. These results are consistent with those previously reported ^[22]^. Combined with the results of Chamberlain et al. ^[17]^ and Schoolmeester et al. ^[18]^, these findings indicate that unlike ASPS, TFE3 overexpression in SST was not caused by genetic translocation, suggesting that other types of genetic alterations may be involved. Chamberlain et al. ^[21]^ proposed that the abnormal accumulation of TFE3 in the nucleus could be caused by the dysfunction of organelles or intracellular metabolic signalling pathways, thus leading to the accumulation of phagosomes typical of granulosa cell tumor of soft tissue (GrCTS) in the cytoplasm. Other studies have reported that TFE3 is involved in the regulation of lysosome/phagosome synthesis and the Golgi stress response ^[22–27]^. Thus, aberrant TFE3 expression can result in degenerative changes due to its lysosomal or cytoplasmic accumulation in these tumours and may not be a unique event for SSTs.

Tumours with abnormal TFE3 expression and gene fusion have some common morphological characteristics, such as abundant cytoplasm and obvious nucleoli. However, SSTs, GrCTSs and other tumours that only express TFE3 without TFE3 gene abnormalities have abundant cytoplasm, but no obvious cell nucleoli. These results indicate that the phenotype of cells is not caused by TFE3 genetic abnormalities.

In conclusion, our results show that TFE3 is expressed in the inner theca cells of normal follicles. Meanwhile, this study also suggests that the immunohistochemical detection of TFE3 is helpful for the diagnosis of difficult cases of sclerosing stromal tumor (such as cystic SST). The Lipid-rich SST cells mimic the inner theca cells of normal ovary and express TFE3 without disrupting the gene structure.

## Figures and Tables

**Table 1:** Clinical findings of SSTs

Patient with SSTs	age	gross	Size (mm)	menstruation	endometrium
Case 1	23	cystic	60	normal	normal
Case 2	17	solid	14	normal	normal
Case 3	26	solid	160	normal	normal
Case 4	26	solid	45	normal	normal
Case 5	39	solid	80	irregular	normal
Case 6	29	solid	60	normal	normal
Case 7	33	solid	50	irregular	atypical hyperlasia
Case 8	23	solid	25	normal	normal

**Table 2:** Immunohistochemical Results of TFE3

Tumor Type	No. of casess	3+	2+	1+	0	Total(%)
Theca cell tumor and theca fibroma tumor	9			2	7	22.2%
Granulosa cell tumor	20				20	0
microcystic stromal tumor	1				1	0
Sclerosing stromal tumor	8	7			1	87.5%
